# Exome sequencing implicates ancestry-related Mendelian variation at *SYNE1* in childhood-onset essential hypertension

**DOI:** 10.1172/jci.insight.172152

**Published:** 2024-05-08

**Authors:** Ian Copeland, Edmond Wonkam-Tingang, Monesha Gupta-Malhotra, S. Shahrukh Hashmi, Yixing Han, Aarti Jajoo, Nancy J. Hall, Paula P. Hernandez, Natasha Lie, Dan Liu, Jun Xu, Jill Rosenfeld, Aparna Haldipur, Zelene Desire, Zeynep H. Coban-Akdemir, Daryl A. Scott, Qing Li, Hsiao-Tuan Chao, Ana M. Zaske, James R. Lupski, Dianna M. Milewicz, Sanjay Shete, Jennifer E. Posey, Neil A. Hanchard

**Affiliations:** 1 Department of Molecular and Human Genetics, Baylor College of Medicine, Houston, Texas, USA.; 2 Childhood Complex Disease Genomics Section, National Human Genome Research Institute, NIH, Bethesda, USA.; 3 Department of Cardiology, Baylor College of Medicine, San Antonio, Texas, USA.; 4 Department of Pediatrics, McGovern Medical School, The University of Texas Health Science Center at Houston, Houston, Texas, USA.; 5 US Department of Agriculture Agricultural Research Service Children’s Nutrition Research Center, Baylor College of Medicine, Houston, Texas, USA.; 6 Baylor Genetics, Houston, Texas, USA.; 7 Human Genetics Center, The University of Texas Health Science Center at Houston, Houston, Texas, USA.; 8 Texas Children’s Hospital, Houston, Texas, USA.; 9 Department of Molecular Physiology and Biophysics;; 10 Division of Neurology and Developmental Neuroscience, Department of Pediatrics; and; 11 Department of Neuroscience, Baylor College of Medicine, Houston, Texas, USA.; 12 Cain Pediatric Neurology Research Foundation Laboratories, Jan and Dan Duncan Neurological Research Institute, Texas Children’s Hospital and Baylor College of Medicine, Houston, Texas, USA.; 13 McNair Medical Institute, The Robert and Janice McNair Foundation, Houston, Texas, USA.; 14 Human Genome Sequencing Center, Baylor College of Medicine, Houston, Texas, USA.; 15 Department of Internal Medicine, McGovern Medical School, The University of Texas Health Science Center at Houston, Houston, Texas, USA.; 16 The University of Texas MD Anderson Cancer Center, Houston, Texas, USA.

**Keywords:** Genetics, Genetic variation, Hypertension, Population genetics

## Abstract

Childhood-onset essential hypertension (COEH) is an uncommon form of hypertension that manifests in childhood or adolescence and, in the United States, disproportionately affects children of African ancestry. The etiology of COEH is unknown, but its childhood onset, low prevalence, high heritability, and skewed ancestral demography suggest the potential to identify rare genetic variation segregating in a Mendelian manner among affected individuals and thereby implicate genes important to disease pathogenesis. However, no COEH genes have been reported to date. Here, we identify recessive segregation of rare and putatively damaging missense variation in the spectrin domain of spectrin repeat containing nuclear envelope protein 1 (*SYNE1*), a cardiovascular candidate gene, in 3 of 16 families with early-onset COEH without an antecedent family history. By leveraging exome sequence data from an additional 48 COEH families, 1,700 in-house trios, and publicly available data sets, we demonstrate that compound heterozygous *SYNE1* variation in these COEH individuals occurred more often than expected by chance and that this class of biallelic rare variation was significantly enriched among individuals of African genetic ancestry. Using in vitro shRNA knockdown of *SYNE1*, we show that reduced *SYNE1* expression resulted in a substantial decrease in the elasticity of smooth muscle vascular cells that could be rescued by pharmacological inhibition of the downstream RhoA/Rho-associated protein kinase pathway. These results provide insights into the molecular genetics and underlying pathophysiology of COEH and suggest a role for precision therapeutics in the future.

## Abstract 2

Exome sequencing in children with early-onset hypertension identifies a putative candidate gene with relevance to ancestry-related disease prevalence.

## Introduction

Childhood-onset essential hypertension (COEH) is a relatively uncommon clinical entity characterized by elevated blood pressures that exceed the 95th percentile for height, age, and sex, without a known (secondary) etiology, in individuals below 18 years of age (1). COEH has an estimated prevalence of 2.1% across ethnic groups in the United States (2). Epidemiological studies show that common contributors to essential hypertension in adults (e.g., diabetes and socioeconomic factors) are much less commonly observed among COEH children, emphasizing the potential contribution of congenital and genetic factors (3). At the same time, COEH confers an increased risk of hypertension in adulthood along with its attendant comorbidities, including stroke, myocardial infarction, and renal disease (4–6). Although COEH affects all ethnic groups, it has been shown to disproportionately affect individuals self-identifying as African American (7–9), who comprise as much as 43% of COEH cohorts (8).

COEH is demographically and clinically distinct from secondary and syndromic forms of childhood hypertension, which are the most common causes of hypertension in children. Secondary causes of hypertension typically result from structural or functional congenital abnormalities of the kidney or vasculature, particularly among premature infants. These defects typically present in the neonatal, infantile, or early childhood period, often with clinical features that extend beyond elevated blood pressure (8, 10–13). COEH also differs strikingly from the adult form of essential hypertension, which is markedly more common (~50%), has a strong environmental component, and has been shown to be polygenic, with hundreds to thousands of common variants collectively contributing to the disease risk (14, 15). In contrast, COEH has a higher estimated heritability than adult-onset essential hypertension (~84%) (16–18), and associated environmental risk factors are less commonly observed. Despite this, the current therapeutic recommendations for adult essential hypertension and COEH are essentially the same, highlighting the dearth of information regarding the molecular pathophysiology in pediatric patients. Unlike genetic studies of adult-onset hypertension, genetic studies of COEH have consisted of small cohorts focused on common variants in candidate genes identified from adult-onset studies (19–23). In most cases, these studies have failed to demonstrate an underlying genetic cause.

Given the comparatively low prevalence of COEH, its high heritability, and the higher genetic burden anticipated of childhood-onset disorders, we hypothesized that a subset of COEH cases might result from the contribution of rare protein-damaging variants segregating in a Mendelian fashion. Other early-onset “adult” diseases, including early-onset Parkinson’s disease (MIM#600116), Alzheimer’s disease (MIM#606889), and diabetes mellitus (MIM#125850), also demonstrate this type of inheritance. Identifying such genes in COEH could provide valuable insight into the underlying pathophysiology and potentially inform efforts at early diagnosis and intervention.

To explore this hypothesis, we undertook exome sequencing (ES) in an extensively well-phenotyped COEH cohort (8). We sought to identify rare, putatively protein-damaging single nucleotide variants (SNVs) and small indels in 16 probands with early-onset COEH but without a strong family history of hypertension. We then leveraged familial segregation, public and in-house sequencing databases, and functional studies of vascular stiffness to identify a putative new COEH gene.

## Results

### Spectrin repeat containing nuclear envelope protein 1 (SYNE1) compound heterozygous, rare, predicted damaging, missense variants are observed in COEH families.

ES was undertaken in 2 groups: group 1 contained 16 families from whom 27 individuals (16 probands, 3 affected siblings, and 8 unaffected family members) were studied by ES. Group 1 individuals were considered to have a higher likelihood of an underlying genetic etiology for their COEH based on observations of an earlier age of onset (median age at diagnosis of 10 years), with either a COEH-affected sibling or a pedigree consistent with an apparently sporadic trait (i.e., no hypertension in either parent). Group 2 consisted of 62 individuals in 48 families (48 probands and 14 unaffected nuclear family members) and was primarily used as a secondary cohort to identify any additional individuals with predisposing variants in high-priority candidate genes from group 1 analyses. All 64 probands and 3 affected siblings had clinically confirmed COEH (8).

**Figure 1:**
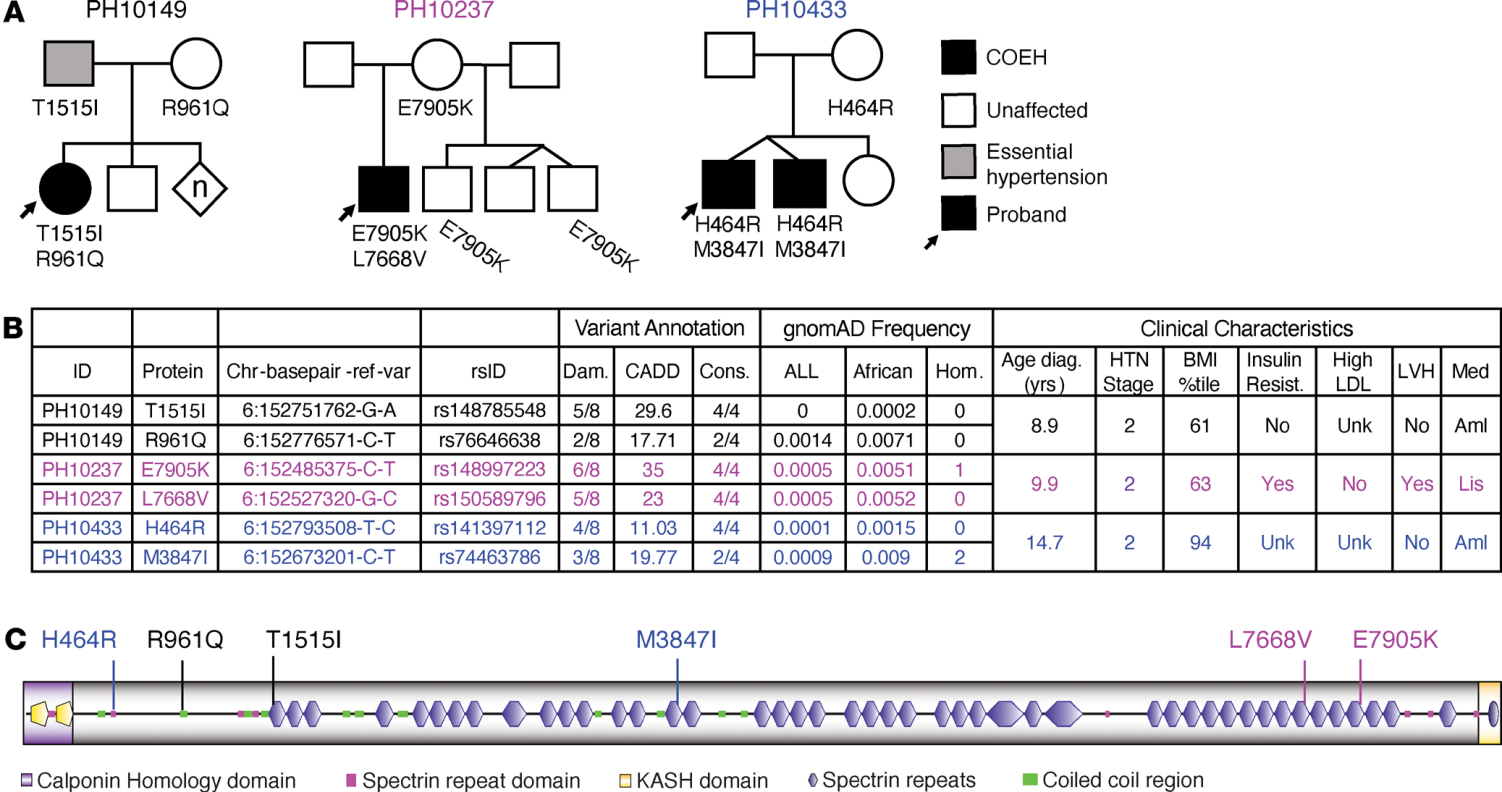
Rare, predicted damaging *SYNE1* variants in COEH probands. (**A**) Pedigrees and variant segregation in affected families. n, multiple individuals of unknown or mixed sexes. (**B**) Variant position, annotation, in silico predictions, and frequencies as well as main clinical features of probands. Chromosome position (Chr) is relative to the hg19 human genome reference. Dam., damaging prediction proportion; CADD, Combined Annotation-Dependent Depletion; Cons., conservation prediction proportion; Hom., number of homozygotes reported; HTN, hypertension; BMI, body mass index; LDL, low-density lipoprotein; LVH, left ventricular hypertension; Med, medications; Aml, amlodipine; Lis, lisinopril. (**C**) Locations of impacted SYNE1 (Nesprin-1) amino acid residues. KASH, Klarsicht, ANC-1, Syne homology.

Our initial analysis focused on identifying rare, nonsynonymous SNVs and small insertions/deletions (indels) in group 1 samples and assessing their familial segregation (Methods). We began by investigating autosomal recessive (AR) and de novo autosomal dominant hypothesized modes of disease inheritance, as these patterns were most consistent with the proband pedigrees (affected siblings and/or unaffected parents). Although we searched the entire ES space (Methods), only 1 gene had multiple SNVs segregating with disease. Specifically, we identified 6 rare, predicted damaging variants in spectrin repeat containing nuclear envelope protein 1 (*SYNE1*) in *trans* (compound heterozygous) in 4 COEH-affected individuals (4/19 affected individuals, ~21%) that segregated with disease in the 3 unrelated families (Figure 1A and Supplemental Table 3; supplemental material available online with this article; https://doi.org/10.1172/jci.insight.172152DS1). Notably, *SYNE1* was also among our priority list of 1,310 genes implicated in hypertension (Supplemental Table 1). These *SYNE1* variants were orthogonally validated and had their segregation verified in available relatives using di-deoxy Sanger sequencing (Methods and Supplemental Figure 1).

Variants identified in our group 1 cohort were rare in current population databases (mean minor allele frequency [MAF] in Genome Aggregation Database [gnomAD] v3.1.2 0.0008; highest MAF 0.0037), putatively protein damaging (median damage prediction proportion > 0.5), and highly conserved (median conservation prediction proportion > 0.8) (Supplemental Table 3 and Methods). All variants were found within the spectrin repeat domain of *SYNE1* (Figure 1C), and at least 1 of the 2 biallelic variants in affected individuals directly affected a spectrin repeat motif. From the remaining 48 group 2 probands, 4 rare, predicted damaging missense variants were identified in 2 unrelated individuals, with each proband carrying 2 variants (Supplemental Table 3). The lack of availability of full trio data for group 2 families made it challenging to definitively assess the phase (in *cis* or in *trans*) of these potentially qualifying variants; we therefore opted to focus on the segregating variants observed in group 1.

Clinically, individuals with biallelic, rare *SYNE1* variants had little evidence of the typical comorbid features associated with COEH. The exceptions to this were 1 proband (PH10433) whose body mass index (BMI) was in the overweight category and another (PH10237) who had both insulin resistance and left ventricular hypertrophy (Figure 1B and Supplemental Table 3). All 3 probands were diagnosed before age 15, and had stage 2 hypertension, requiring either a calcium channel blocker (PH10433 and PH10149) or an angiotensin-converting enzyme inhibitor (PH10237) (Figure 1B). From the medical history and physical examination, none of these 3 probands had clinical evidence of neurological, muscular, or skeletal disease or a history of heart attack or stroke (Supplemental Table 3).

### Rare, biallelic SYNE1 variation is enriched among individuals with COEH.

**Figure 2:**
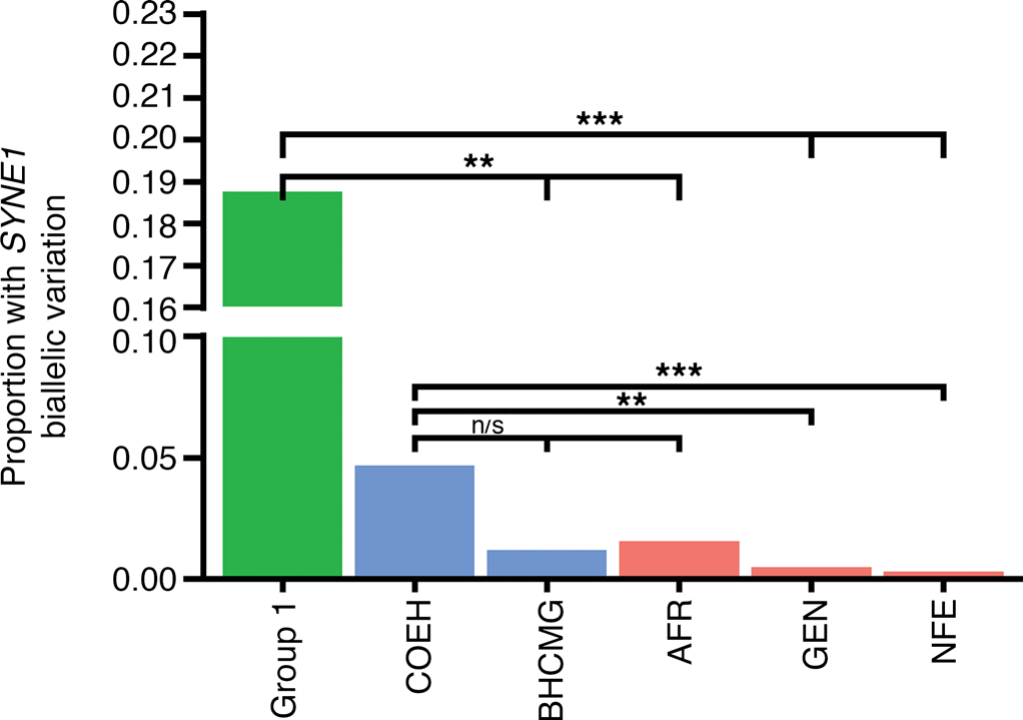
Comparison of biallelic *SYNE1* missense proportions. Statistical comparisons were performed using a binomial test. **P < 0.01; ***P < 0.001. Group1, group 1 of COEH cohort (*n* = 16 probands); COEH, full COEH cohort (*n* = 64 probands); BHCMG, Baylor-Hopkins Center for Mendelian Genomics; AFR, gnomAD self-reported African samples; GEN, gnomAD combined samples; NFE, gnomAD self-reported non-Finnish European.

*SYNE1* is a large gene, comprising 515,716 nucleotides and 146 exons at its longest transcript; thus, rare *SYNE1* variation is not uncommon in ES data. To provide additional confidence for the specificity of our observations and verify that our result was not simply the result of chance variation in a large gene, we used public and in-house databases to assess the frequency of biallelic *SYNE1* variation in cohorts that were not enriched for COEH. As neither family nor phase information was readily available in gnomAD, we chose to calculate the probability of identifying 2 rare variants in *trans* from the frequency distribution of rare (MAF < 0.01), missense *SYNE1* variants for each population ancestry group (Methods). When we compared these values with the proportion of unrelated probands with confirmed *SYNE1* biallelic variation (group 1 = 3/16, 18.8%; COEH cohort = 3/64, 4%), group 1 and COEH cohort individuals had significantly higher proportions of putative *SYNE1* damaging variation (group 1 or COEH vs. ancestry groups *P* < 0.01), and this was true regardless of population ancestry (Figure 2).

To achieve a more accurate estimation of biallelic variation and assess the impact of differences in sequencing platform and bioinformatic pipelines between our data and gnomAD, we also evaluated *SYNE1* biallelic variation in a data set of 1,700 parent-proband trio-ES from the Baylor-Hopkins Center for Mendelian Genomics (BHCMG) cohort (Methods). We identified 20 of 1,700 probands (1.2%) with rare, predicted damaging, compound heterozygous, missense variants in the spectrin repeat domain of *SYNE1*. Of these probands, only 1 individual reported mixed African ancestry, described as both African/Caribbean and European; the remainder of reported ancestries were European or Asian. This frequency was significantly lower than observed in our group 1 individuals (*P* < 0.01, Fisher’s exact test) and lower, though not significantly so, for the combined COEH cohort (Figure 2). These data suggest that rare, putatively protein-damaging, missense variation in *trans* in *SYNE1* is enriched in our COEH cohort.

Next, we queried the Baylor Genetics (BG) cohort using a reverse genetics approach to explore blood pressure in carriers of rare, putatively damaging, compound heterozygous variants in the spectrin domain of *SYNE1* who were not in our COEH cohort (Supplemental Figure 3, Methods, and Supplemental Table 4). None of the recruited individuals met clinical criteria for a diagnosis of hypertension, though, as a group, the median diastolic blood pressures were above the 50th percentile. BG cohort individuals were of similar age (median ages of 9.9 for the COEH cohort and 11.5 at last measurement of the BG cohort) but were generally shorter (median height percentiles BG 14.5, range 1–46; and COEH 33, range 24–88), heavier (median BMI percentiles: BG — 96, range 2–98; COEH — 63), and of self-reported European ancestry. Unlike in our COEH cohort, blood pressures in the BG cohort were single measurements done at routine (nonhypertensive) clinic visits and were not obtained using recommendations from the Fourth Report (1).

Last, we sought to use the All of Us (AoU) database to further contextualize our variant findings, as AoU includes more than 18,000 individuals with pediatric electronic health records (EHRs) (Methods). To do so, we assessed the proportion of individuals with EHR-derived COEH carrying *SYNE1* damaging variants. We identified 104 cases that met criteria for an International Classification of Diseases–based (ICD-based) diagnosis of childhood hypertension and had data from short-read whole-genome sequencing (srWGS). We then created a control group of 10,939 individuals with srWGS data but without a hypertension diagnosis in their EHRs. Consistent with previous studies, we found participants with genetically predicted (per AoU) African and Latino/admixed American ancestries to be the most represented among COEH cases, each accounting for 35% of cases (Supplemental Table 5A). We first queried the numbers of carriers of our 6 candidate variants in both cases and controls. Out of the 104 cases, we found 2 individuals each carrying a different variant (Supplemental Table 5B). In the controls, the carrier rate was approximately 0.3% for each of the 6 candidate variants. For all AoU participants with srWGS data, the reported MAF ranged from 0.006% to 0.3% (Supplemental Table 5B), making the 6 candidate variants extremely unlikely to be seen in the 104 cases. When we considered the entire spectrin repeat domain, none of the cases or controls carried a loss-of-function variant, and the proportion of cases that were heterozygous for rare, putatively damaging, missense variants was slightly higher than, but not significantly different than, that observed among the 10,939 controls (cases 24%; controls 21%; *P* = 0.5, χ^2^ test). Four cases (3.8%) had more than 1 *SYNE1* damaging variant, compared to 3.1% (343/10,939) of controls (*P* = 0.6, χ^2^ test), though the phase of these variants could not be determined (Supplemental Table 5C).

### Rare, damaging SYNE1 missense variation is more common among individuals with African genetic ancestry.

**Figure 3:**
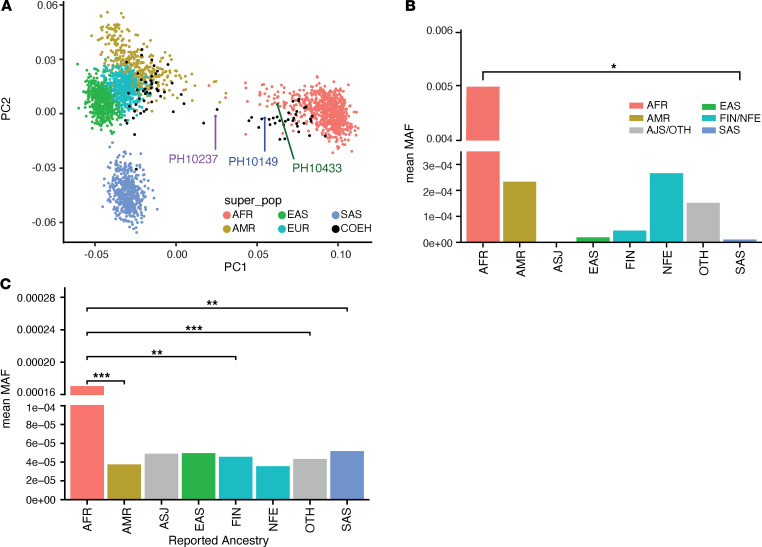
Population ancestry studies. (**A**) Principal component analysis (PCA) of ES-derived genetic ancestry for “super-populations” (super_pop) from the 1000 Genomes Project (AFR, African; EAS, East Asian; SAS, South Asian; EUR, European; AMR, Latino/Admixed American) and COEH. *n* = 64 (probands with *SYNE1* variants are labeled). (**B**) Mean minor allele frequencies (MAFs) among gnomAD populations of 6 putative disease variants identified in COEH. *P < 0.05. (**C**) MAFs of all *SYNE1* rare and damaging missense variants in gnomAD. ***P < 0.001; **P < 0.01. Ethnicity abbreviations are per gnomAD: AFR, African; AMR, Latino/Admixed American; ASJ, Ashkenazi Jewish; EAS, East Asian; FIN, Finnish European; NFE, non-Finnish European; SAS, South Asian. Statistical significance in **B** and **C** was determined by 1-way ANOVA.

COEH is known to have a higher incidence and prevalence among individuals self-identifying as African American (2, 9). Remarkably, every individual harboring compound heterozygous *SYNE1* variants in the COEH cohort self-reported being of African descent. This observation was consistent with individuals’ genetic ancestry, ascertained via principal component analysis (PCA; Figure 3A and Methods). This observation raises the intriguing hypothesis that ancestry-enriched variation may play a role in the heightened prevalence of COEH among individuals of African descent.

To evaluate this further, we first examined the mean allele frequencies of the 6 putative COEH variants found in our COEH families. In gnomAD, each of these variants had a significantly higher mean MAF in the African population group (Figure 3B). We then calculated the mean MAF of all rare (MAF < 0.01), missense, and putatively damaging (CADD score > 12) *SYNE1* variants in gnomAD. The mean frequencies of rare variants in *SYNE1* were highest among individuals of reported African ancestry and significantly higher than other ancestry groups available for comparison (Figure 3C). This relatively increased frequency of gnomAD rare variants was not observed in either of 2 other linker of nucleoskeleton and cytoskeleton (LINC) complex genes — lamin A/C (*LMNA*) and SUN domain-containing protein 1 (*SUN1*) (Supplemental Figure 4) — and is not known to be a general feature of African ancestry populations.

### Decreased SYNE1 expression leads to increased vascular smooth muscle cellular stiffness that can be rescued by fasudil.

**Figure 4:**
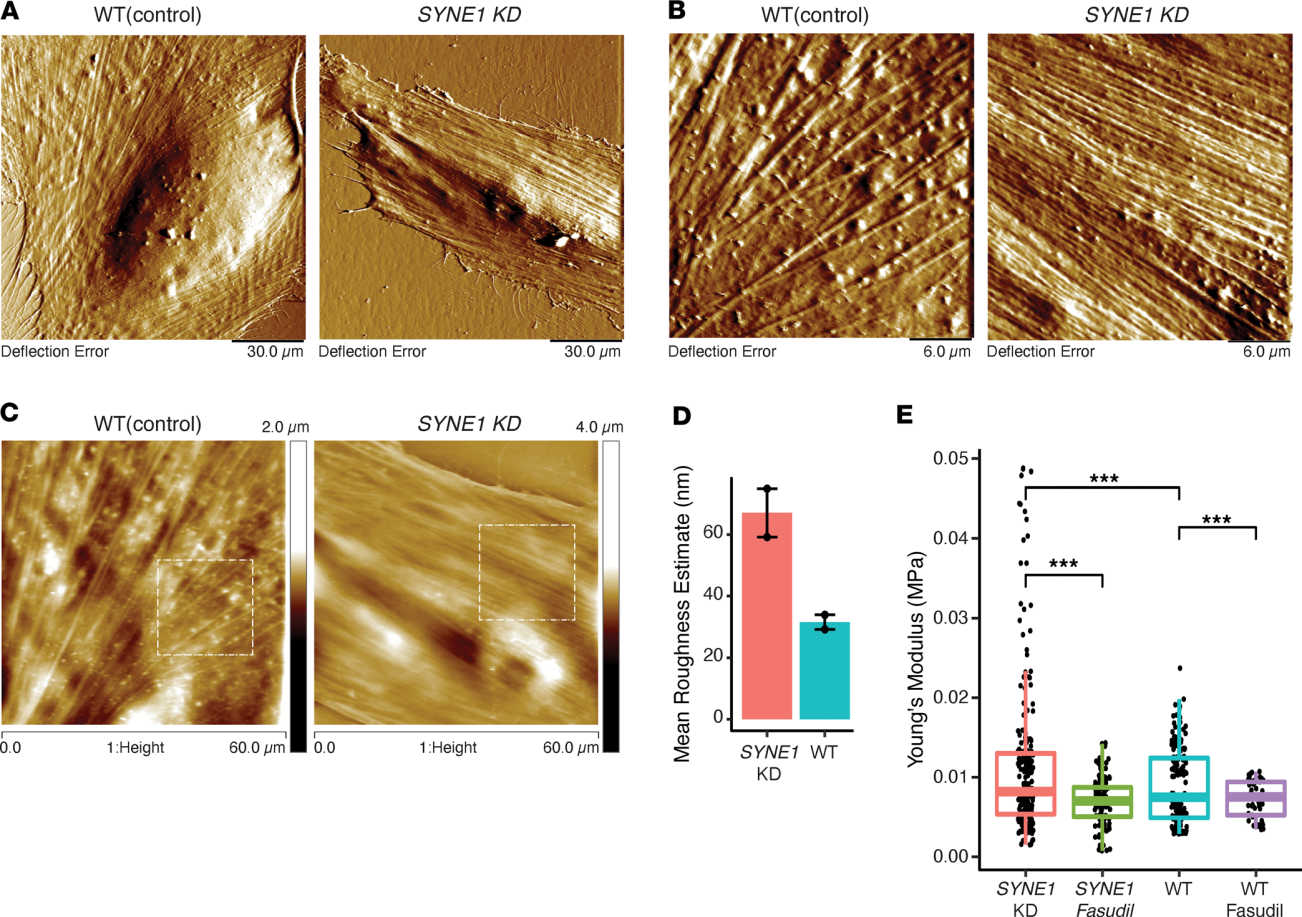
*SYNE1* functional studies. Low- (**A**) and higher (**B**) magnification scans to 130 and 30 μm (*x*-*y*), respectively, showing detailed topography of control and *SYNE1*-KD cells from atomic force microscopy imaging. (**C**) Height images (60 μm^2^ scans) were used to evaluate roughness within a 20 μm (*x*-*y*) measuring box to define a specific area of analysis (400 nm^2^) in each cell. (**D**) Roughness estimates between *SYNE1*-KD cells and control (wild-type) cells (*n* = 2 each), quantifying presumed actin filament formation. (**E**) Comparison of stiffness (measured as Young’s modulus) in control (wild-type) cells, *SYNE1*-KD cells, and both wild-type and *SYNE1*-KD cells treated with fasudil. Box plots show the interquartile range, median (line), and minimum and maximum (whiskers). Statistical comparisons were performed using 1-way ANOVA. ***P < 0.001.

*SYNE1* is highly expressed in cardiac, skeletal, and vascular smooth muscle tissues (24), and common variants near *SYNE1* have been associated with pulse pressure and mean arterial pressure in 2 large GWAS of hypertension. Therefore, aberrant expression of *SYNE1* is a strong candidate to affect vascular integrity (25, 26). We hypothesized that recessive inheritance of a putatively damaging variation might lead to reduced functionality of the resulting *SYNE1* protein, Nesprin-1, which could affect vascular stiffness and lead to hypertension. To evaluate this contention, we used atomic force microscopy (AFM) — the gold standard for measuring the elasticity of living cells — to compare the elasticity of control (wild-type) vascular smooth muscle cells (VSMCs) versus VSMCs with decreased *SYNE1* expression as a result of RNA interference knockdown (KD) of *SYNE1* (Methods) (27). We found that the mean expression of *SYNE1* in *SYNE1*-KD VSMCs was 2-fold lower than in control cells (Supplemental Table 6), and this was accompanied by a visible increase in cell striation (Figure 4, A–C) and increased roughness (Figure 4D) in *SYNE1*-KD VSMCs. Quantitatively, *SYNE1*-KD VSMCs had significantly more stiffness (lower elasticity) (*P* = 0.003) than control VSMCs (Figure 4E and Supplemental Table 7) (28).

Increased RhoA/Rho-associated protein kinase (ROCK) activity has been noted to inhibit vascular stiffness in *SYNE1* loss-of-function models (29). Ablation of LINC genes, such as *LMNA* and *SYNE1*, in immortalized human myoblasts reduces expression of ROCK and MYH9 (29) and thereby reduces ROCK phosphorylation, ultimately resulting in increased stress fiber accumulation and contraction in VSMCs (30). We thus hypothesized that fasudil, a pharmaceutical agent (HA-1077) that selectively inhibits ROCK (31) and has been approved for clinical use in Japan and China (32), could ameliorate the effect of *SYNE1* KD in VSMCs. To assess the effect of fasudil in our in vitro model, we treated both control and *SYNE1*-KD cells with fasudil and measured cellular elasticity with AFM. Fasudil-treated *SYNE1*-KD cells (*SYNE1* *FA*) had a significantly lower mean elasticity score than *SYNE1*-KD cells (*P* = 3 × 10^–7^) that was also comparable to that from control cells (*P* = 0.16) (Figure 4E and Supplemental Table 7).

## Discussion

Hypertension in children is difficult to definitively diagnose and, historically, was not as closely evaluated or followed-up in routine pediatric visits as recommended. Thus, large cohorts of patients with COEH or of families with multiple affected members are unusual, and genetic studies of “nonsyndromic” COEH are uncommon in the literature. Perhaps as a result, to the best of our knowledge, there are no other genes definitively implicated in COEH. Here we report genetic, physiological, and biophysical experimental evidence supporting the contention of a role for *SYNE1* in the development of early-onset hypertension. We identified 6 population-rare, putatively damaging, missense variants in *SYNE1* segregating in a compound heterozygous manner in approximately 20% of individuals with COEH. All affected individuals were young children and adolescents and had no evidence of a known genetic syndrome. We show that the number of compound heterozygous *SYNE1* variants in our cohort significantly deviates from observed and theoretical expectations (Figure 2) and that this variation is enriched among individuals of African genetic ancestry. Finally, we provide biophysical experimental support for the functional role of reduced *SYNE1* in the vascular stiffness phenotype seen in hypertension.

Given the challenges of identifying children with true COEH, our study population represents a uniquely large and comprehensively characterized cohort. Compound heterozygous, putatively damaging, missense *SYNE1* variants were found among individuals who were largely devoid of associated comorbidities (obesity, insulin resistance) and diagnosed before age 15. These individuals did not have a strong family history of adult-onset hypertension or overt evidence of an underlying syndrome. This profile, wherein individuals with the most extreme or most atypical phenotypes have strong evidence for a genetic contribution to disease, is consistent with early-onset forms of other adult diseases.

In our cohort, putatively damaging, compound heterozygous *SYNE1* variants were observed in individuals self-reporting as African American, which comprise 47% of our COEH cohort, and rare variation in this gene appears to occur more frequently among individuals of African genetic ancestry. The prevalence and incidence of essential hypertension in adults are higher among individuals identifying as African American, and this phenomenon has been associated with an increased incidence of lifestyle and environmental risk factors in this group. Compared with other ethnic groups, the incidence of COEH among self-identifying African Americans appears to be independent of classical environmental risk factors (3, 8). Our observations suggest a role for rare *SYNE1* variation in the increased predisposition for COEH among Americans of African ancestry and bolster the expanding recognition that African genomes can inform human migration and health (33).

The *SYNE1* protein — Nesprin-1 — is a large protein comprising 3 primary domains: the spectrin repeat domain, the calponin homology domain, and the KASH domain. Spectrin repeats are abundant in the primary isoform of *SYNE1* expressed in smooth muscle vascular cells, whereas the KASH domain is expressed only among isoforms found in neural tissue. Biallelic, loss-of-function *SYNE1* variants in the calponin homology and KASH domains have been implicated in AR spinocerebellar ataxia-8 (MIM#610743) and arthrogryposis syndrome arthrogryposis multiplex congenita 3, myogenic type (MIM#618484), whereas rare missense and rare truncating *SYNE1* variants within the spectrin repeat domain have been implicated in an autosomal dominant form of Emery-Dreifuss muscular dystrophy (MIM#612998) (34, 35). Identified variants in our cohort all occurred within the spectrin repeat domain that makes up the majority of Nesprin-1, and all probands had at least 1 of their biallelic variants directly affecting a spectrin repeat motif. These results are consistent with a model of allelic heterogeneity among *SYNE1*-related rare disease traits, where the specific motifs and domains affected, alongside allele-specific gene dosage, e.g., biallelic versus monoallelic, contribute to downstream clinical consequences.

Murine knockouts of *Syne1* have been used to explore the role of *SYNE1* in cardiac and skeletal muscular disease (36, 37). The International Mouse Phenotyping Consortium (38) reported that *Syne1*-knockout mice exhibit an incompletely penetrant preweaning lethality, but none of these models had blood pressure assessments. Mouse models may not always accurately recapitulate a human disease trait, especially as complete loss of function may not model human biallelic variant combinations. Nesprin-1 was originally isolated and characterized in a search for novel markers for VSMC differentiation (39), and it functions within the LINC complex (29, 40). The LINC complex scaffolds the nucleus and interacts with the cytoskeleton directly, influencing muscle cell nuclear morphology (29), mechanotransduction (41), and muscle cell elasticity (41). Genes encoding LINC complex proteins are also implicated in the development of Emery-Dreifuss muscular dystrophy (29, 42–44), and rare variants in LINC complex genes have been implicated in congenital cardiovascular diseases, including thoracic aortic disease and dilated cardiomyopathy (45–47). Taken together, these observations further support the contention for *SYNE1* variation as a molecular contributor to COEH.

Individuals with comparable *SYNE1* variation in the BG cohort did not have definitive evidence of COEH, suggesting that biallelic *SYNE1* variation may show incomplete penetrance in COEH. It is possible that other genetic or environmental factors (e.g., salt intake), including ancestry- or age-specific ones, may be needed for disease manifestation (48). Similarly, it may be that filtering variants solely based on bioinformatics features does not ensure a like-to-like match in pathogenic potential. It is also worth noting that individuals in the BG cohort were of self-reported European ancestry, and the assessments of blood pressure were limited to isolated, outpatient, automated and manual measurements. ES in these individuals was undertaken as part of an evaluation for rare Mendelian diseases, including developmental delay and congenital abnormalities, which is partly reflected in the wide range of BMI and height centiles in this group. Therefore, we cannot dismiss the potential for these individuals to develop hypertension later or to still have underlying, undiagnosed hypertension. We also note that in the context of Mendelian diseases the inability to replicate findings does not necessarily constitute rebuttal evidence, as candidates might be private or specific to an environment or population. This is a particular consideration for populations of African ancestry, who have deep ancestral histories with variable exposures over time and a high level of genetic diversity (33).

The demographics of the 104 cases identified in AoU matched those noted in previous COEH studies (~35% among individuals of genetically predicted African and Latin/admixed American ancestry, respectively) (49), though we did not observe a statistically significant difference between COEH cases and nonhypertensive controls in the proportion of individuals with putative deleterious *SYNE1*. As a caution against extrapolating too much from these observations, however, we note that the pediatric EHR controls are generally younger adults (presently) and are likely to include undiagnosed or unreported hypertensive individuals; only 17% had an adult diagnosis of hypertension, compared with the reported approximately 50% globally (15) and across the full AoU cohort. Our preliminary foray in AoU also illustrates the challenges in using EHR data for childhood-onset diseases and traits, especially those with variable age of onset and progression.

Our cell-based functional assay showed that reduced expression of *SYNE1* in VSMCs leads to increased cell stiffness, which may correlate with increased vascular stiffness and resistance. There is a scarcity of data in the literature to establish a presumed pathophysiologic mechanism of COEH; speculatively, in the context of presumed large-effect Mendelian inherited variants, reduced *SYNE1* expression in our probands could be associated with sufficient vascular stiffness and downstream vascular resistance to overwhelm the endogenous regulatory mechanisms (e.g., vasodilation, reduced renin secretion) designed to correct increased vascular resistance. Alternatively, the increased vascular resistance presumably induced by higher vascular stiffness could reduce perfusion to effector organs, including the kidney, which could activate the renin/angiotensin/aldosterone pathway, leading to a worsening of vasoconstriction with excess sympathetic tone and aldosterone secretion, as seen in renovascular hypertension (50). This latter potential emphasizes the need for future studies to also assess the metabolic changes associated with vascular stiffness in the context of COEH.

We have identified biallelic, rare variation within *SYNE1* among young children with COEH. The likely damaging nature of these variants paired with the observed effect of *SYNE1* KD in vascular in vitro models suggests that aberrant production of *SYNE1* may ultimately lead to increased total peripheral resistance and subsequently hypertension. Our data also allude to a potential loss-of-function model in COEH individuals with compound heterozygous *SYNE1* missense variation. Aside from the identification of a strong COEH candidate gene, our data are a starting point for considerations of clinical sequencing among individuals with COEH and provide a framework for genetic studies of COEH cohorts, which could collectively herald new paradigms for diagnosis and treatment in the future.

## Methods

### Sex as a biological variable.

Recruitment of human participants for this study included both sexes without discrimination. Sex was not used as a separate biological variable, as each family unit is expected to demonstrate independent Mendelian segregation of variants on autosomal chromosomes, to which the analysis was limited.

### Human participant recruitment and phenotyping.

The details of the cohort have been reported (3, 8). Briefly, 423 individuals were referred to a pediatric hypertension clinic at McGovern Medical School at The University of Texas Health Science Center at Houston (UTHealth), Texas Medical Center. Participant ages ranged from 0 to 19 years. Hypertensive status was confirmed by use of manual auscultation with a mercury sphygmomanometer in all COEH probands. All individuals underwent 24-hour ambulatory blood pressure monitoring to assess systolic and diastolic blood pressure levels per diagnostic recommendations from the Fourth Report (1, 8). Hypertension was assessed based on the individual’s height, age, and sex and was reported as systolic and diastolic blood pressure percentiles. As recommended by the Fourth Report, gestational age, blood and urine tests, renal ultrasound and magnetic resonance imaging, echocardiogram, and a sleep study in obese individuals were used to rule out secondary forms of hypertension. Renin, aldosterone, and angiotensin levels were also used to assess potential secondary/monogenic forms. From this previously established cohort, blood, urine, and DNA were obtained from 64 probands and 300 affected and unaffected family members.

### ES.

ES was carried out at the Human Genome Sequencing Center (HGSC) at Baylor College of Medicine (BCM) in 2 separate phases, corresponding to 2 groups — group 1 contained 16 families and 27 individuals (16 probands, 3 affected siblings, and 8 unaffected family members) and represented the probands with early disease onset and no history of COEH in preceding generations; group 2 consisted of the remainder of the cohort (48 probands). The SeqCap EZ HGSC VCRome 3 capture library, developed by Roche NimbleGen, was used to capture approximately 23,585 protein-coding genes (including exons and intronic flanking sequences) and 189,028 nonoverlapping exons. The average depth of coverage of sequencing was ~100×–120×, with 95% of the human exome covered at >20×. Resulting FASTQ files were aligned to the hg19 build of the human genome using Burrows-Wheeler Aligner with variant call files (VCFs) rendered using the Mercury pipeline, which is an automated pipeline for the processing of FASTQs, including quality control, mapping, exclusion of sequencing artifacts, and variant calling using Atlas2 (51–54).

### Annotation and quality control.

VCFs were annotated using Variant Tools 2.7.0 and Varcards (55, 56). VCF tools version 4.0 was used to impose a depth of coverage filter of >10× and a “PASS” filter to exclude low-quality sites (57). The HGSC’s parameters for PASS notation include a Q30 score distribution > 80%, PASS filter > 60%, and PhiX control error rate < 2%. A size cutoff of 15 bp was used when evaluating small indels. An allelic ratio cutoff of >4:10 was used when evaluating all variants.

The database for human nonsynonymous SNPs and their functional predictions were used to identify likely loss-of-function and missense variant alleles and to exclude synonymous variants (58). Eight functional prediction algorithms were used to characterize missense variants: Sorting Intolerant From Tolerant (SIFT), likelihood ratio test (LRT), MutationTaster, Mutation Assessor, Polymorphism Phenotyping v2 (Polyphen2), Functional Analysis through Hidden Markov Models (FATHMM), Protein Variation Effect Analyzer (Provean), and CADD (59–72). We then determined a damage prediction proportion for each variant that reflected the proportion of algorithms for which a given variant was denoted as putatively damaging, which included denotations of “damaging,” “possibly damaging,” “probably damaging,” “disease causing,” “deleterious,” and “medium,” and CADD scores > 12. Genomic Evolutionary Rate Profiling (GERP), phylogenetic p-values (phyloP), SIte-specific PHYlogenetic analysis (SiPhy), and PHylogenetic Analysis with Space/Time models (phastcons) were also used to assess evolutionary conservation of a particular variant across species. By convention, strong conservation was determined by scores >12 for SiPhy, >4.4 for GERP, >1.6 for phyloP, and >0.5 for phastcons (73–77). Each variant was then given a final “conservation prediction proportion,” the proportion of conservation algorithms supporting strong evolutionary conservation.

### Prioritization of candidate variants.

Following VCF annotation, candidate variants were identified by using gnomAD data to exclude variants with MAF > 0.01. gnomAD (version 2) is a publicly available data set that includes variant data from 15,708 genomes and 125,748 exomes (78). Most pedigrees examined were supportive of either an AR or de novo dominant disease model, and a model of full penetrance of candidate variants was used. De novo variants were reviewed in families with available sequencing data from both parents and were considered when present in the proband and none of the parents. The Kinship-based INference for Genome-wide association studies (79) was used to confirm paternity and/or maternity within each family.

A list of 1,310 genes associated with various forms of hypertension was compiled from Model organism Aggregated Resources for Rare Variant ExpLoration, GWAS catalog, OMIM, and publications implicating genes in monogenic forms of secondary hypertension and vascular diseases (Supplemental Table 1) (10, 11, 80–85). This gene list was used to further prioritize candidate variants.

### BHCMG.

The BHCMG exome variant database was used as both a sequencing and non-COEH control cohort to assess the frequency and segregation of candidate variants sequenced on the same platform. The BHCMG database at BCM consists of 10,244 exomes including 1,700 parent-child trios (at the time of analysis). Individuals within this cohort are all suspected to have Mendelian disease traits and were sequenced at the HGSC using similar library capture, sequencing platform, and annotation software (86, 87). We compared the difference in the proportion of individuals with rare, predicted damaging, missense *SYNE1* biallelic variation (in *trans*) between the COEH and BHCMG cohorts. To make the analysis true to the variants identified in our COEH cohort, all variants satisfied a damage proportion and conservation proportion score of ≥0.25 and ≥0.5, respectively.

### Probability assessment of biallelic variation in gnomAD.

We compared the proportion of individuals with rare, predicted damaging, missense *SYNE1* biallelic variation (in *trans*) identified in group 1 and group 2 in the COEH cohort to the probability of observing rare, damaging, missense *SYNE1* biallelic variation (in *trans*) in gnomAD. To make the analysis consistent with variants identified in our COEH cohort, included variants had to have a damage proportion and conservation proportion score of ≥0.25 and ≥0.5, respectively, and impact the spectrin repeat domain of the resulting protein. Significance was denoted by *P* < 0.05. The proportion of *SYNE1* biallelic variation (in *trans*) in gnomAD was estimated by assessing the probability of any individual having at least 1 rare, missense variant in *SYNE1*. We then used this parameter as the starting point to simulate mating of 2 individuals where both are heterozygous for at least 1 rare missense variant in *SYNE1* and both variants are passed to 1 offspring under an assumption of Hardy-Weinberg equilibrium and an outbred population. We squared the values of the probability of each parent having at least 1 rare missense mutation and multiplied this by 0.25 (see equation below) to give the probability of having in *trans* variation in *SYNE1*. This result produced probabilities for biallelic, rare *SYNE1* variation for all (combined) ancestries and for each individual ancestry group in gnomAD. *P* = (0.25) (*B*^2^) *B* = (*A*) (1 – *A*) *A* = (1 – *f*_1_)…(1 – *f*_n_)

*P* represents the probability of biallelic inheritance, *A* represents the product of the frequencies (*f*) of the reference (major allele) for all rare variants (*n*) within the gene, *B* represents the product of the cumulative variant frequencies across the gene in question, and *f* represents the frequency of a given rare variant (with MAF < 1%) in the gene in question.

### BG cohort.

We identified individuals with rare and damaging missense variants in *SYNE1* using available trio data from the BG cohort. A subset of the sequence data in the BG cohort is from individuals being evaluated for suspected Mendelian/genetic disorders at the Pediatric and Adult Genetics clinics at BCM and Texas Children’s Hospital. To make the analysis comparable, we limited inclusion criteria to individuals with biallelic variants, where both variants satisfied a damage proportion and conservation proportion score of ≥0.25 and ≥0.5, respectively. Individuals with the appropriate genotypes were then invited through their Texas Children’s Hospital/BCM physicians to have their blood pressure measurements, age, and height included in the study after provision of informed consent. Blood pressure measurements were taken from the EHRs and primarily reflected single standard-of-care measurements taken manually or via automated cuff at the time of outpatient clinic visits. As is customary with individuals referred for diagnostic testing to BG, the primary referring physicians for most individuals were neurologists or medical geneticists (88).

### AoU database initiative.

We attempted to utilize the AoU Research Program initiative (89, 90) to explore the potential for using large-scale databases for COEH, as AoU includes approximately 18,870 individuals with available pediatric EHR data. From the AoU database, we constructed a cohort of cases and controls. We defined cases of COEH as participants with an ICD-based diagnosis of hypertension (ICD10CM: 35207668) before the age of 18 years and excluded from those participants with no recorded blood pressure measurements, with no srWGS data, and with history of a potential secondary etiology of hypertension before the diagnosis (this includes kidney disease, pregnancy within 3 months prior to the diagnosis, and intake of medications that can potentially increase blood pressure). The control group was made of individuals with available srWGS data and no record of childhood hypertension in their pediatric EHR or diagnosis of hypertension as adults. We then performed a gene burden test, considering rare (MAF < 1% in gnomAD) and damaging variants (loss of function and deleterious missense) occurring within the spectrin repeat domain of *SYNE1*, and compared proportions between cases and controls.

### Assessment of genetic ancestry.

COEH probands with compound heterozygous variants in SYNE1 were genotyped on the Infinium H3Africa Consortium Array v2. Multidimensional scaling was used to assess the ethnicity of COEH individuals and compare them with super-population ancestries in the 1000 Genomes phase III data (91), which were downloaded from https://ftp.1000genomes.ebi.ac.uk/vol1/ftp/release/20130502/ The downloaded.vcf files were converted into PLINK 1 binary format, filtering out the non–A-T and non–G-C SNPs. A set of SNPs with MAF > 0.20 were selected from the merged COEH cohort ES data and the 1000 Genomes data set (*N* = 82,508). Linkage disequilibrium pruning (--indep 50 5 1.1) implemented in PLINK (92) v1.9 was used on the selected SNPs prior to the PCA.

### Database comparison of allele frequencies across ethnic groups.

All rare (MAF < 0.01) and damaging (CADD score > 12) missense gnomAD variants collected till March 2022 were acquired through PopViz (https://hgidsoft.rockefeller.edu/PopViz/), a web-based program designed to aggregate and visualize gnomAD data (93). Protein domain architecture information was acquired from SMART web resource (http://smart.embl-heidelberg.de/) (94, 95). Rare, damaging variants located on the *SYNE1* domain were used for the comparison. The remaining variants were then stratified by ethnic group, and allele frequencies across groups were compared in a pairwise manner.

### Validation of candidate variants.

Candidate variants were validated using di-deoxy Sanger sequencing of PCR-generated amplicons of 200–300 bp. Amplicon primers were generated in silico using Primer Plus and purchased from MilliporeSigma (Supplemental Table 2). Amplification was performed on a DNA Engine Tetrad 2 thermocycler (Bio-Rad) (96). To investigate variant segregation, validated candidate variants were genotyped using di-deoxy Sanger sequencing from whole-blood DNA of available family members who had not previously undergone ES. Genotypes of validated variants were called and visually confirmed from Sanger chromatograms using Sequencher 5.4.6 (Gene Codes Corporation) (Supplemental Figure 1).

### Culture of VSMCs.

VSMCs were acquired from Coriell Institute for Medical Research, catalog number AG11548. This cell line was derived from the iliac artery of a 17-year-old cadaveric donor without known COEH or hypertension. VSMCs were at 36 passages when acquired. Cells were initially grown in collagen-treated 60 mm dishes (Corning catalog 354401), then transferred to 100 mm Corning Biocoat Gelatin Cellware (catalog 354653). At 50% confluence, cells were then transferred to T-75 gelatin-coated flasks. Vasculife basal medium (Part LM-0002) was used for nourishment.

### Cell preparation for live elasticity measurements.

We prepared 60 mm Petri dishes coated with Rat Tail Collagen I (Thermo Fisher Scientific, A1048301, at a concentration of 50 μg/mL) for AFM live-cell scanning. Cells were cultured to a 50% confluence and probed in cell culture medium.

### Cell preparation for roughness analysis.

Cells were seeded in collagen-coated (50 μg/mL) 60 mm polystyrene plates to 50% confluence and incubated for 24–48 hours at 37°C in a 5% CO_2_ atmosphere. Media were removed and cells were fixed with 2 mL of paraformaldehyde 4% for 15 minutes before scanning (see scanning method in the paragraph below). All cell culture work was performed by the Tissue Culture Core at BCM, which is a clinical translational core laboratory for the Department of Molecular and Human Genetics.

### AFM scanning.

AFM was conducted in the Atomic Force Microscopy Facility at the UTHealth. Data were collected using a BioScope II Atomic Force Microscope (Bruker) integrated with a Nikon TE2000-E inverted optical microscope. Force curves from at least 15 randomly chosen cells per treatment were registered using Novascan colloidal AFM probes. These probes consisted of a 5 μm diameter borosilicate glass particle attached to the edge of a silicon nitride V-shaped cantilever with a nominal spring constant of 0.24 N/m. The cantilever was calibrated for its laser sensitivity using the thermal oscillation method before each experiment. Indentation curves were captured using 4 μm ramp sizes, corresponding to an indentation depth of approximately 40 nm, a scan rate of 0.5 Hz, and a trigger threshold with a maximum load of 10 nN. Young’s modulus was calculated in live cells following the Hertz model (spherical indenter radius = 2.5 μm) with a Poisson’s ratio of 0.5, using the NanoScope Analysis software (version 1.5, 2015, Bruker). The mean levels of elasticity measurements were compared between control VSMCs (control cells), *SYNE1*-KD VSMCs, and *SYNE1* *FA* cells. Topographical images were taken on cells fixed with 4% paraformaldehyde and scanned in PBS. The structure of the cell membrane was determined using contact mode operated in liquid to a scan rate of 0.7 Hz. Images were captured to a scan size ranging from 30 to 130 μm (*x*-*y*). Random cells were selected, with the aid of optical microscopy (original magnification, 20×). Fixed cells were scanned in PBS using MLCT cantilevers (*fo* = 4–10 kHz, *k* = 0.01 N/m, ROC = 20 nm) from Bruker. The roughness of the cell membrane was determined by isolating an area (20 × 20 μm) in the cytoplasm, away from the cell nucleus in the 60 μm (*x*-*y*) scan, and comparing the same area (400 nm^2^) in all the cell conditions (Figure 4C). Topographical roughness was calculated as the “arithmetic average of the absolute values of the surface height deviations measured from the mean plane” as defined in the NanoScope Analysis software (version 1.5, 2015, Bruker).

### Lentiviral infection and SYNE1 KD.

RNA interference using shRNA was used to reduce *SYNE1* transcription using a pGIPZ lentiviral vector from Dharmacon. In brief, lentivirus was packaged in HEK293T cells integrated into the vector system purchased from Dharmacon using pGIPZ control and *SYNE1* shRNA clones. Viruses were collected 48 hours posttransfection. Cells were plated at approximately 30% density in 6-well plates the day before infection, and the media were changed 24 hours after infection. Cells were then treated with puromycin (2 μg/mL) for 3 days before further analysis. Successful infection was assessed qualitatively via GFP fluorescence using confocal microscopy (Supplemental Figure 2). Control cells were infected with lentivirus encoding the empty vector (without shRNA targets). Lentiviral packaging and infection were conducted at the Cell-Based Assay Screening Service core at BCM.

*SYNE1* KD was confirmed via quantitative reverse transcriptase PCR. RNA extraction of VSMCs was done using the RNeasy Mini Kit (QIAGEN). Extractions were conducted as 6 separate biological replicates. Briefly, the cells were trypsinized, collected, suspended, and lysed. The lysate was then homogenized using 350 μL of RLT lysis buffer followed by vortexing and ethanol elution. RNA buffer RW1 (700 μL) was used as a washing agent to purify RNA followed by final elution of whole RNA in 50 μL of RNase-free water. Concentration of RNA was quantified using NanoDrop (Thermo Fisher Scientific) and Bioanalyzer (Agilent) with resulting RNA integrity numbers > 9. Whole RNA was stored at –80°C until cDNA generation using the OneStep RT PCR Kit (QIAGEN) according to manufacturer’s recommendations using 10 μL of RNA per sample. Reverse transcriptase PCR was conducted using a TaqMan assay probe (Thermo Fisher Scientific) targeted to the first exon of *SYNE1*, which is present in all *SYNE1* mRNA transcripts. Amplification was performed on a DNA Engine Tetrad 2 (Bio-Rad), initially for 30 minutes at 50°C to initiate reverse transcription, followed by 15 minutes at 95°C to activate the DNA polymerase. The samples were then cycled for 40 cycles under PCR conditions of 1 minute at 94°C, 1 minute at 68°C (annealing), 1 minute at 68°C (extension), and 10 minutes at 72°C and run on a ViiA 7 Real-Time PCR System (Thermo Fisher Scientific). A minimum cycle quantification value > 15 was obtained for the 3 replicates performed. Relative expression was quantified using the Livak method (97).

### Drug treatment.

*SYNE1*-KD and control (wild-type) cells were treated with 3 mL of fasudil (10 mg suspended in 7 mL of sterile water) (Santa Cruz Biotechnology) added to the cellular media. After 30 minutes’ incubation at room temperature, media (containing fasudil) were removed by aspiration.

### Statistics.

R software was used for statistical analysis, and statistical significance was denoted by *P* < 0.05 after correcting for multiple testing (where appropriate, in most cases a Bonferroni correction for the number of tests). Fisher’s exact test was used to compare the proportion of individuals with rare, damaging, missense *SYNE1* variants between the COEH and BHCMG cohorts. A binomial test was used to compare the proportion of individuals with rare deleterious *SYNE1* biallelic variation (in *trans*) in the COEH cohort to the predicted proportion with similar variation (i.e., rare, damaging *SYNE1* variation in *trans*) in gnomAD. For the AoU database, the χ^2^ test was used for proportion comparison between cases and controls. A 1-way ANOVA test was used to compare allele frequencies of *SYNE1* rare, damaging variants across ethnic groups in gnomAD and evaluate the difference in the mean level of elasticity measurements obtained from the AFM between cell types (i.e., controls, *SYNE1* KD, fasudil treated). For the specific case of elasticity data, statistical significance was denoted by a *P* < 0.01.

### Study approval.

All data and materials from human participants were obtained in accordance with research parameters approved by the Institutional Review Board at BCM, the Institutional Committee for the Protection of Human Subjects, or the Institutional Review Board at the UTHealth and Children’s Memorial Hermann Hospital, Texas Medical Center (all in Houston, Texas, USA).

### Data availability.

Data supporting this manuscript are available in the supplement and the Supporting Data Values file.

## Author contributions

NAH and MGM conceived the study design. MGM, SSH, DMM, and SS recruited the participants and collected, collated, and analyzed the clinical data. IC, EWT, YH, AJ, and NL conducted the genomic data analyses. EWT, ZD, and QL performed the AoU data analysis. IC, NJH, and AH performed quality control of samples, DNA amplification, and DNA sequencing. PPH processed and prepared the tissues; DL and JX performed the lentiviral transfection KD experiments; and AMZ conducted the AFM experiments. DAS and HTC conducted the clinical reviews for the reverse phenotyping of the BG cohort. JRL, ZHCA, JR, and JEP coordinated and conducted the analyses of the BHCMG data. YH and AJ conducted and drafted figures for the ancestry and gnomAD analyses and assisted with statistical comparisons. IC, NJH, AH, and EWT performed the confirmatory validations. IC, JEP, and NAH drafted the first version of the manuscript; EWT and NAH wrote and drafted subsequent versions. All coauthors read, edited, and approved the final submitted manuscript. The order of the co–first authors was based on the length of involvement of individual authors in the project, with respect to the primary and secondary manuscript drafts.

## Supplementary Material

Supplemental data

Supplemental tables 1-7

Supporting data values

## Version 1 05/08/2024

Electronic publication
