# Supplementary material for: Exome sequencing implicates ancestry-related Mendelian variation at SYNE1 in childhood-onset essential hypertension
Source: JCI Insight. 2024 May 8;9(9):e172152. doi: 10.1172/jci.insight.172152 (PMC11141928; doi:10.1172/jci.insight.172152)
Supplement: Supplemental data [file jciinsight-9-172152-s037.pdf]

Supplementary Figures

Supplementary Figure S1 - Di-deoxy (Sanger) chromatograms

Supplementary Figure S2 – Stable Knockdown of SYNE1

Supplementary Figure S3 - Reverse phenotyping of CEOH carriers in BHCMG

Supplementary Figure S4 – Comparisons of LINC gene minor allele frequencies between ancestry groups

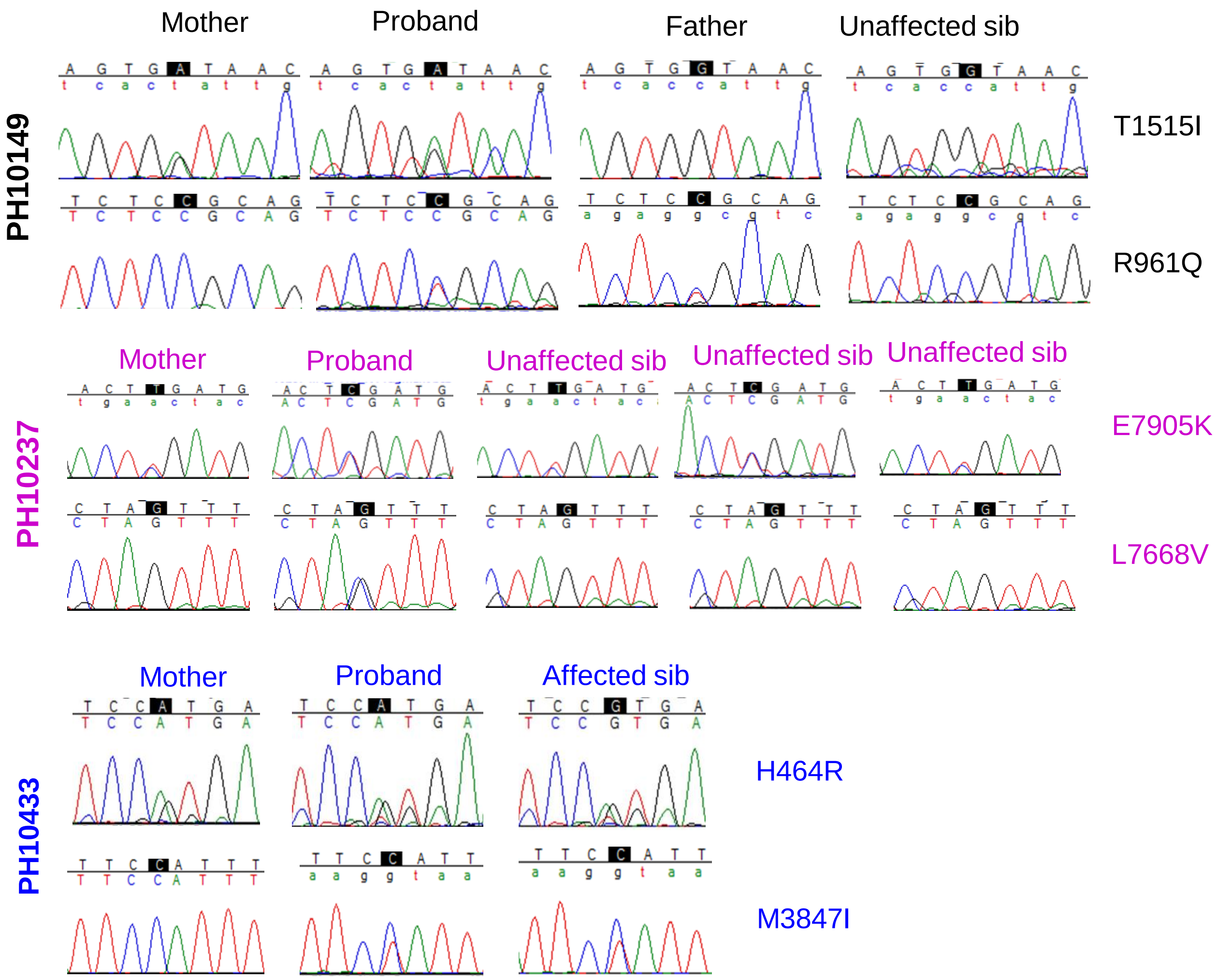

Supplementary Figure S1: Di-deoxy (Sanger) chromatograms of confirmed *SYNE1* variants. Missense SNVs for each family (indicated by PHXXXX) are shown to the right of each chromatogram set.

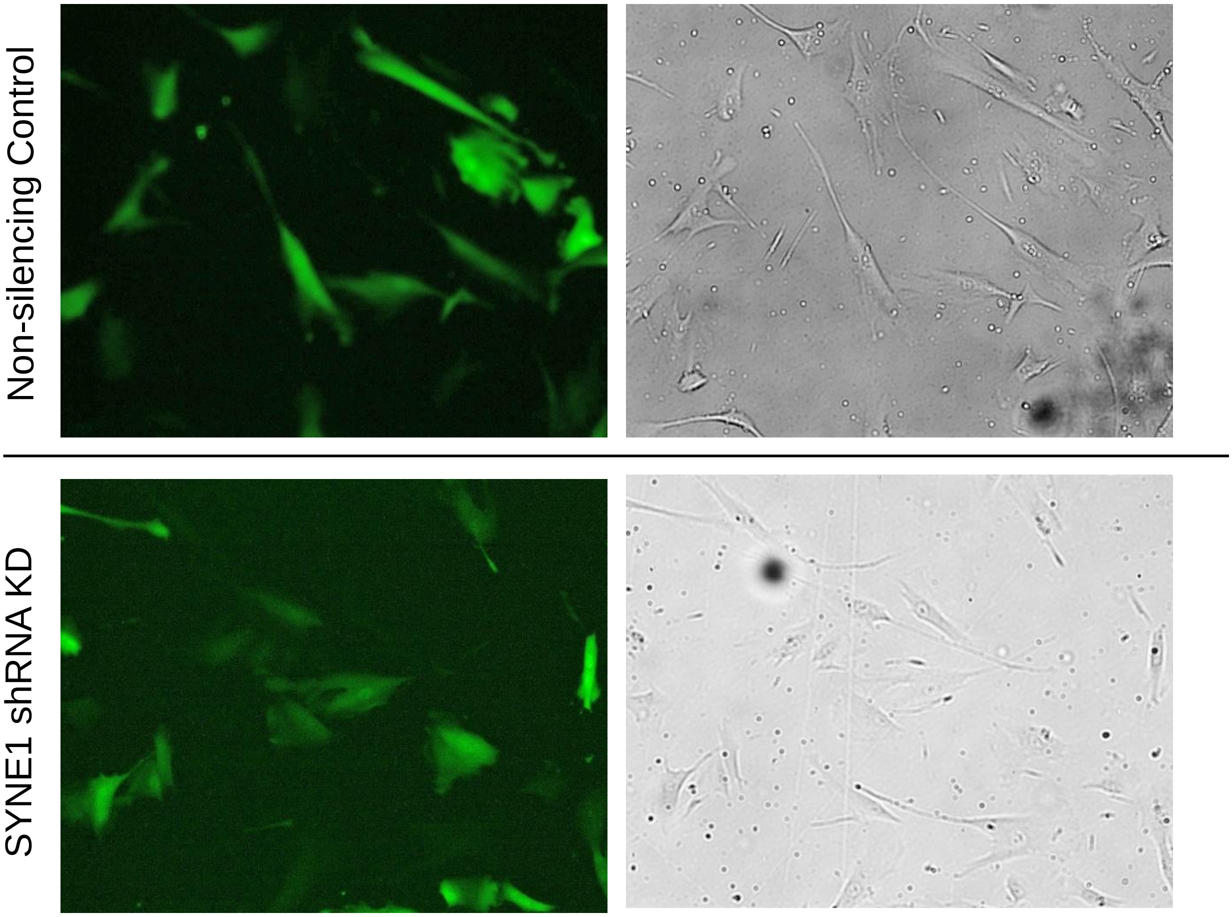

Supplementary Figure S2: Stable knockdown (KD) cells showing near 100% expression of GFP, indicating successful transduction with the shRNA viruses.

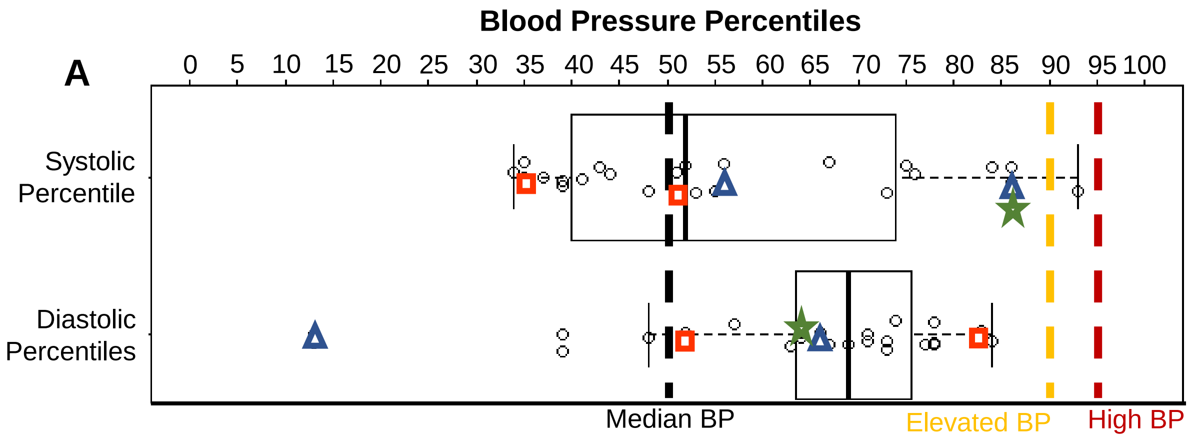

B

Symbol	AApos	Sex	Age	BMI	CADD	DP	Allele
△	D388N	M	12	-	32	0.75	Het
	P2278L			-	25	0.875	Het
○	R1395W	M	11	97	31	0.625	Hom
□	L2679P	M	7	4	26	0.875	Het
	C3662Y				13	0.5	Het
★	D4566	M	9	97	32	0.75	Het
	L8547F				32	0.875	Het

Supplementary Figure S3: A. Median systolic/diastolic percentiles for BG individuals with biallelic rare and damaging variants in *SYNE1.* B. Variant level and demographic information for individuals with *SYNE1* compound heterozygous variation identified in the BG.

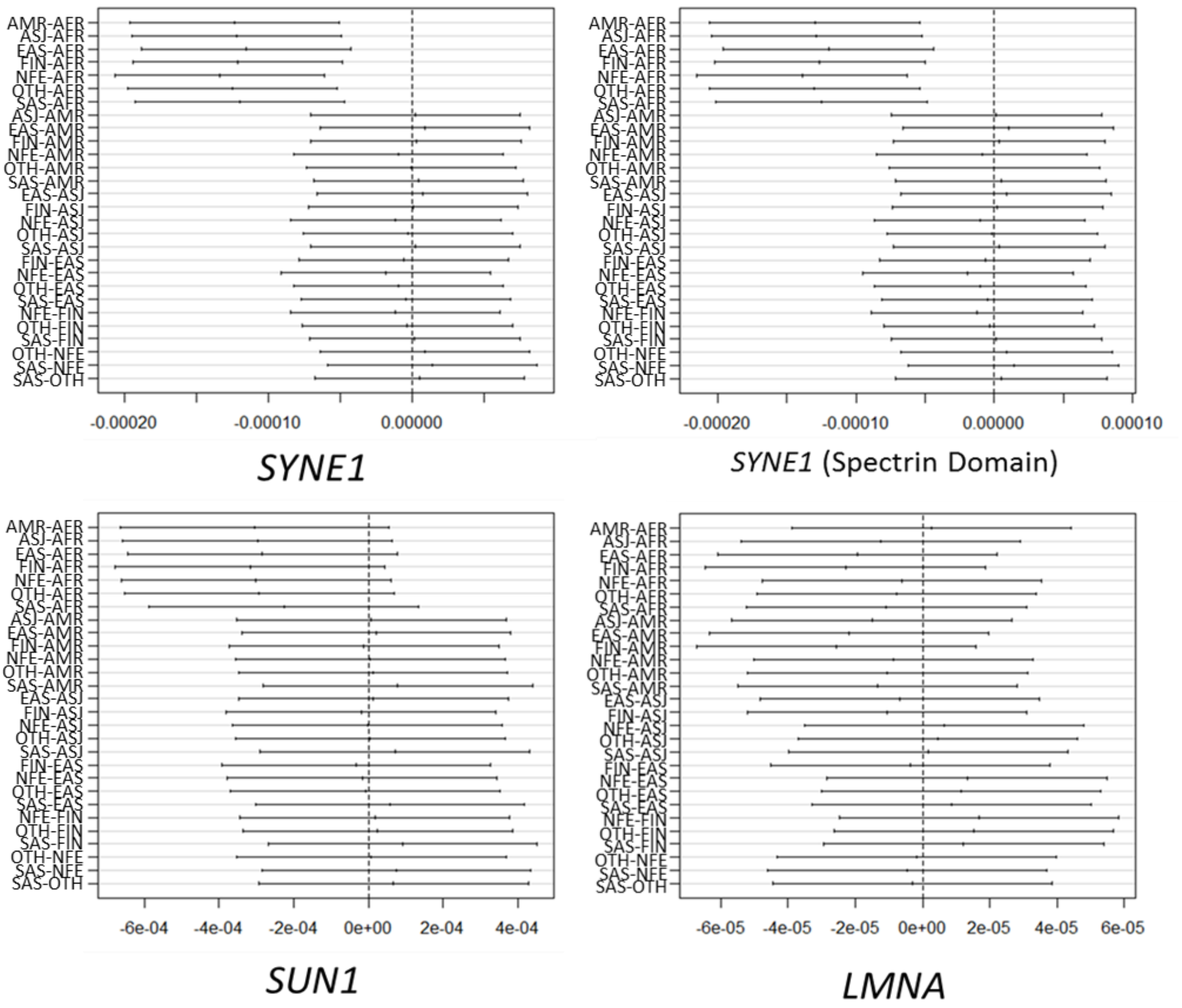

Supplementary Figure S4: Anova test results (Tukey HSD) of minor allele frequency (MAF) from rare damaging missense variants in *SYNE1*, *SYNE1* (spectrin domain only), *SUN1* and *LMNA*. Whiskers show 95% confidence intervals. Where confidence intervals contain zero (indicated by the dotted line) group comparisons are not considered significant.
